# Effects of Fe Staple-Fiber Spun-Yarns and Correlation Models on Textile Pressure Sensors

**DOI:** 10.3390/s22093152

**Published:** 2022-04-20

**Authors:** Minki Choi, Chi Cuong Vu, Jooyong Kim

**Affiliations:** Department of Organic Materials and Fiber Engineering, Soongsil University, Seoul 06978, Korea; yc5424@textra.or.kr (M.C.); cuongvc287@gmail.com (C.C.V.)

**Keywords:** spacer fabric, textile-structure, pressure textile sensor, metallic yarns, correlation model

## Abstract

As an aspect of intelligent clothing, e-textile sensors can flexibly sense and transmit information about human bodies and environments. However, difficulties relating to their technology and the variation in textile materials employed in their manufacture still limit their ability to analyze and be applied. The authors’ previous publication deployed a pressure sensor with warp-knitted spacer fabrics, wet-knitted fabrics, Ag-yarns, and Fe-yarns. An equivalent circuit analyzed the resistance behavior with some effects of the Ag-coated twisted yarns. In the present paper, the authors continue to evaluate the correlation model R-ε and the effects of the Fe staple-fiber spun yarns in detail. Together, the two studies provide an extensive understanding of the textile-related elements that affect pressure sensors. In addition, the process and the analysis (correlation model) could bring the textile sensors here developed close to the manufacturing stage, particularly for high precision/adjustable applications. We also develop a simple touch sensor matrix to demonstrate the potential of the sensor and the analyzing method.

## 1. Introduction

E-textile sensors are a strong research direction and have excellent prospects in the future due to the ease with which they may be approached and applied. By adding electronic mechanisms, textile sensors can be used in wearable devices [1,2,3,4,5,6], human–machine interfaces [7,8,9], or controlling/monitoring applications [10,11,12,13]. There are many benefits of textile materials: air permeability, drape, lightweight, or elasticity. These properties can give an intelligent garment suitability, ease of use, and high productivity [14,15,16,17].

The structural factors of the textiles, which can be designed, are called the “tailorability” feature. Considering the tailorability of pressure textile sensors, we can divide these into two structures. Typical pressure sensors are multilayered consisting of two electrode layers and a sensing layer in the middle [18,19,20]. However, it is challenging to control performance by designing the textile structures. In other words, these sensors have low reproducibility and non-tailorability [21,22,23]. Another structure is the sensing yarns or fibers. In this case, there is no laminating process (no multilayering). Resistance changes under deformations. This type has a small thickness, so the step-production is also reduced. The effects of the textile elements now become complex and the tailorability is low. In addition, the yarn sensors need some coating or dipping processes.

Previous studies have reported on textile sensors made from many different materials and processes. For instance, Kim et al. [24] developed a hand-drawing force sensor with pyralux film and conductive fibers by dipping them in carbon nanotube inks (CNTs). Their sensor is thin, has a high performance, and can detect touch in water. An et al. [25] proposed an MXene-textile pressure sensor for industrial and biomedical applications based on a typical dipping-drying method. The MXene-textile sensor achieved a response time of 149 ms, a low detection limit of 219 mN, and good sensitivity of 19 kPa^−1^. In other research, Kim et al. [26] used 3D spacer textiles, carbon nanotube inks, and drop-coating methods to create high-performance pressure sensors. These spacer textile sensors showed a wide range of sensing capability of 200 Pa–50 kPa and excellent durability of up to 10,000 cycles. However, their studies only focus on the materials or fabrication processes without analyzing, in detail, the textile or fiber structures.

To improve the existing issues of the above structures, we propose an analyzing method from the correlation model (R-ε) on the pressure textile sensors. The sensors are composed of Fe staple-fiber spun yarns, warp-knitted spacer fabrics, and Ag-coated twisted yarns. The resistance changes through the slope of the conductive yarns under pressure. The transmission of the signals is mainly dependent on the characteristics and structures of the e-textile elements.

## 2. Materials and Methods

As in the authors’ previous study [1], five structures of the warp-knitted spacer fabric (Wp-Sfa) were obtained from Dongwoo Fiber Co. Ltd., Seoul, South Korea. There are differences in the thickness, density, and diameter of the pile yarns of each fabric (Figure S1). The spacer fabric is an actual 3D fibrous structure and includes two layers at the bottom/top and an interconnect layer of yarns oriented in the third dimension. The Fe staple-fiber spun yarns (*Fe-sp*) were prepared from VCTEC Co. Ltd., Seoul, South Korea (Figure S2a). The Ag-coated twisted yarns (*Ag-tw*) were twisted double at 10,000 rpm by an X-static 70 Dienie machine from Noble Biomaterials Inc., United States (Figure S2b).

A plane-binding structure of the sensors [1] has two layers of the conductive wet-knitted fabrics (*Wt-Cfa*) at the bottom and top, which act as electrode layers. The fabric includes multi-PET yarns and *Ag-tw* fibers, created from a circular knitting machine (Topnit KCPJ-III) with 96 feeders and 8 rpm speed. This knitting machine has a gauge of 28 and a diameter of 36 inches from Kem Young Co., Ltd., Seoul, South Korea. The *Wt-Cfa* fabric has a total of 9.6 conductive courses per 8 mm and 14 non-conductive courses per 12 mm.

These yarns have a diameter of 302 µm and conductivity of 7.77 Ω/cm. These were obtained by a KC250B-Twist machine from Textile-Machinery Co., Ltd., China. In addition, the knitted-sensing yarns (*Fe-sp*) connect two *Wt-Cfa* layers at three different slope directions: x/y-slope, x-slope and no slope. The angle of inclination is 5.2°, and the number of *Fe-sp* is 4, 16, and 36. The *Fe-sp*s warp and make the contact thereby creating changes in electrical resistance when under pressure.

## 3. Results and Discussion

### 3.1. Mechanical Properties

Figure S3a shows a universal testing machine with an LCR meter (Keysight E980AL) and a force load cell (DN-FGA-20). The parameters are set up at the speed of 0.02 mm/s and the resolution of 0.0098 N/cm^2^. Figure S3b–d show the working performance of the sensor types: x/y-slope, x-slope, and no slope. We found that the x-slope or no-slope samples have low homogeneity in the buckling direction and the x/y slope samples have a high degree of homogeneity.

The pile yarns are designed with an inclination angle of 5.2° for each slope, and these angles secure the operation of the sensors as the aim and equivalent circuit diagram. With no-slope samples, we predict four buckling directions, including two in opposite and two in the same directions. The no-slope or x-slope types are complicated to analyze and need more experiments in the future.

To determine the operational phases, we represent the buckling contacts in the parallel structures of the resistors [1]. In the first bending phase, the resistance changes according to the appearance of the primary contacts (*R_pri-contact_*). The contact resistance is a variable that appears between the sensing yarns (Fe staple-fiber yarns). This value is high before loading pressure (when the Fe-yarns are not in contact) and becomes small under loading pressure. This resistance is close to 0 when the sensing yarns are in contact. In the second bending phase, the interference expands and leads to the increase in the secondary contacts (*R_sec-contact_*). Other phases (3rd, 4th, etc.) also generate new contacts by the same mechanism. As a result, total thickness reduces, and the area in which electrodes can move is continuously shrunk. Eventually, the overall resistance changes.

From the result of the previous study [1], we can calculate the resistance change in different phases and the strain-stress correlation from Equation (1), where$\;R_{0}$ is the initial resistance of the stitched *Fe-sp*, $n_{thrd}$ is the number of the *Fe-sp*, $h^{\prime }$ is the height of side, and h is the thickness of fabric (stage 0).

(1) $$\in =\frac{h^{\prime }}{h}\;,\;R^{\prime }=R_{\left(Fe-SpStitch\right)}^{\prime }=\frac{R_{0\left(Fe-sp\right)}}{{\left(2n_{thrd}\right)}^{2}}h\left(1-\in \right)$$

### 3.2. Dynamic Model of the Warp-Knitted Spacer Fabrics

Two primary features of the warp-knitted spacer fabric (Wp-Sfa) are the structural characteristics and the number of pile yarns. Table 1 shows some factors of the Wp-Sfa, including the thickness (*h*), the diameter of the pile yarns ($d_{pll})$, the length of the pile yarns ($l_{pll}$), the number of the pile yarns ($n_{pll}$), the curvature radius of the pile yarns (*R*), the center angle of the curvature (∠β), AB straight length, and DB straight length. In addition, the tensile modulus ($E_{pll\left(ex\right)}$) is the tensile stress per diameter area of the pile yarn and is calculated from Equation (2).

(2) $$E_{pll}=E_{pll\left(ex\right)}A_{pll}n_{pll}=E_{pll\left(ex\right)}{\left(\frac{d_{pll}}{2}\right)}^{2}\pi n_{pll}$$

Considering the buckling of the pile yarns (Wp-Sfa), we divide this dynamic behavior into four stages. The first and second stages could be predicted by using the structural factors and Chen’s model [27]. Equations (3) and (4) show the models of the two first stages:

(3) $$\sigma \left(\epsilon \right)=\frac{\sigma_{1}}{\epsilon_{1}}\epsilon =\left[\frac{\left\{\pi RE_{pll}r_{pll}\sin\left(\frac{90l}{\pi R}-\alpha \right)\right\}h}{4r_{1}^{3}\left\{h-2R\sin\left(\frac{90l}{\pi R}-\alpha \right)\right\}}\right]\epsilon$$

where $r_{1}$ is the central angle of the pile yarn curve in stage 1; and

(4) $$\begin{array}{l} \sigma \left(\epsilon \right)=\frac{\sigma_{2}}{\epsilon_{2}}\left(\epsilon -\epsilon_{1}\right)+\epsilon_{1}\\ \;\;\;\;\;\;\;=\left[\frac{h\left(\pi E_{pll}r_{pll}^{4}\right)}{\{h-2R\sin\left(\frac{90l}{\pi R}-\alpha \right)-\left(r_{1}\sin w+r_{1}\sin\frac{\beta_{1}}{2}\right)\}4r_{2}^{2}}\left(\epsilon -\frac{h-rR\sin\left(\frac{90l}{\pi R}-\alpha \right)}{h}\right)\right]\\ \;\;\;\;\;\;\;+\frac{\pi RE_{pll}r_{pll}\sin\left(\frac{90l}{\pi R}-\alpha \right)}{4r_{1}^{3}} \end{array}$$

where *ω* is the ∠A501F in stage 2 and $r_{2}$ is the radius of the pile yarn bending circle in stage 2.

The third and fourth stages are difficult to predict by the geometric method. However, we can analyze these stages through the phenomenology of cellular solids [22].

(5) $$\sigma \left(\epsilon \right)=A\left(1-e^{\left(-\frac{E}{A}\epsilon^{a}{\left(1-\epsilon^{b}\right)}^{c}\right)}\right)+e^{m}\left(e^{\epsilon^{n}}-1\right)$$

**Figure 1:**
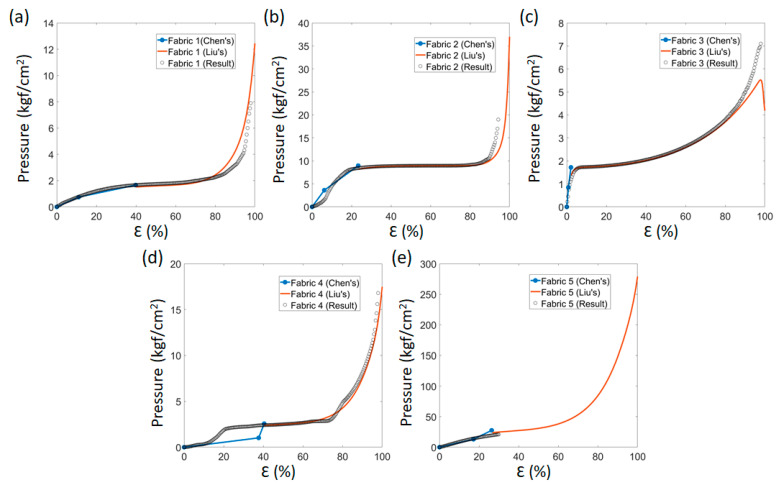
Comparison of the values from the predicted models (Chen’s and Liu’s model) and actual data of (**a**) Fabric 1, (**b**) Fabric 2, (**c**) Fabric 3, (**d**) Fabric 4, and (**e**) Fabric 5.

Liu et al. [28] determined the relationship between structural components of the fabric and seven undefined variables (*A*, *E*, *a*, *b*, *c*, *m*, *n*). Of these, *A* is the same as the stress magnitude at the endpoint of the 2nd step, and *E* is proportional to the modulus. Excepting *A*, the other variables can only be obtained from actual values and from fitting graphs (Figure 1).

In the secondary phase, the resistance behaviors, as generated from the contacts, can be expressed by the thickness of the deformed sensor and the strain–resistance correlation equation:

(6) $$\epsilon =\frac{h^{\prime }}{h}\;,\;R^{\prime }=R_{\left(Fe-spStitch\right)}^{\prime }=\frac{R_{0\left(Fe-sp\right)}}{{\left(2n_{thrd}\right)}^{2}}h\left(1-\epsilon \right),\;\mathrm{but}\;\left[\frac{4h-\sqrt{4h^{2}-3g_{thrd}^{2}}}{3h}\right]<\epsilon <1$$

We obtained the final correlation model between resistance and pressure by substituting the above equation into the dynamic behavior model (Equation (5)).

(7) $$\sigma \left(R\right)=A\left[1-e^{\left[-\frac{E}{A}{\left(1-\frac{R_{\left(Fe-spStitch\right)}^{\prime }{\left(2n_{thrd}\right)}^{2}}{R_{0\left(Fe-sp\right)}h}\right)}^{a}\right]{\left\{1-{\left(1-\frac{R_{\left(Fe-spStitch\right)}^{\prime }{\left(2n_{thrd}\right)}^{2}}{R_{0\left(Fe-sp\right)}h}\right)}^{b}\right\}}^{c}}\right]+e^{m}\left(e^{{\left(1-\frac{R_{\left(Fe-spStitch\right)}^{\prime }{\left(2n_{thrd}\right)}^{2}}{R_{0\left(Fe-sp\right)}h}\right)}^{n}}-1\right)\;\mathrm{but}\;\frac{4h-\sqrt{4h^{2}-3g_{thrd}^{2}}}{3h}\leq \epsilon \leq 1$$

where $n_{thrd}$ is the number of *Fe-sp*, $R_{0\left(Fe-sp\right)}$ is the initial resistance of *Fe-sp*, and {*A*, *E*, *a*, *b*, *c*, *m*, *n*} are Liu’s model variables.

### 3.3. Effects of the Fe Fiber Spun Yarns

**Figure 2:**
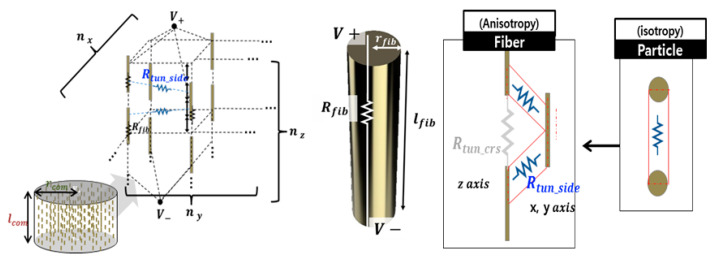
The resistance of the composite materials is calculated by the tunneling resistance model [27].

According to Oskouyi et al. [29], the resistance of the composite materials is calculated by the tunneling resistance model. Assuming no fiber-to-fiber contacts, the electrons moves in the tunnel inside the fibers. We therefore connect the conductive parallel fibers in the direction of the current flows (Figure 2). This arrangement can transform into a model of initial resistance (Equation (8)).

(8) $$R_{0\left(Fe-sp\right)}=\left(\frac{l_{thrd}}{{\left(d_{thrd}\right)}^{2}\pi }\sqrt[{3}]{\frac{V_{fib}}{Q_{0}}}\right)\left\{\left(\rho_{fib}\frac{l_{fib}}{{\left(\frac{d_{fib}}{2}\right)}^{2}}\right)+\left(\frac{4.94\left\{10^{23}e^{\left(4.1\lambda \sqrt{\lambda }\right)}\right\}}{\left(\frac{d_{fib}}{2}\right)\left(\frac{l_{fib}}{2}\right)\sqrt{\lambda }}\sqrt{2}\left(\sqrt[{3}]{\frac{V_{fib}}{Q_{0}}-2r_{fib}}\right)\right)\right\}$$

where $d_{thrd}$ is the diameter of *Fe-sp*, $l_{thrd}$ is the length of *Fe-sp*, $Q_{0}$ is the volume fraction of conductive staple fiber, $V_{fib}$ is the volume of conductive staple fiber, $d_{fib}$ is the diameter of conductive staple fiber, $l_{fib}$ is the length of conductive staple fiber, $\rho_{fib}$ is the resistivity of conductive staple fiber, and *λ* is the energy barrier.

From image analysis, we found the total diameter $(d_{thrd}$) of the spun yarn was 287 μm. The diameter ($d_{fib})$ and the length ($l_{fib}$) of the short conductive fibers were 13.75 μm and 980 μm, respectively. From XRF analysis, we determined the weight ratio of the entire yarn (*Fe-sp*) was 16.57% of Fe, 3.27% of Ni, and 80.16% of PET (Figure S2b). In addition, the calculated resistance (using Equation (8)) is 9.86 kΩ, and the actual measured value is 10.32 kΩ. The penetrated structure of *Fe-sp* determines the initial resistance. This value can be used to ascertain the length of *Fe-sp*. Therefore, the resistance of Wp-Sfa (with penetrated *Fe-sp*) can be calculated in Equation (9).

(9) $$R_{0\left(Fe-spStitch\right)}=\frac{h}{{\left(2n_{thrd}\right)}^{2}}R_{0\left(Fe-sp\right)}\;,\;g_{thrd}=\frac{\omega_{fabric}}{{\left(2n_{thrd}\right)}^{2}}\;$$

**Figure 3:**
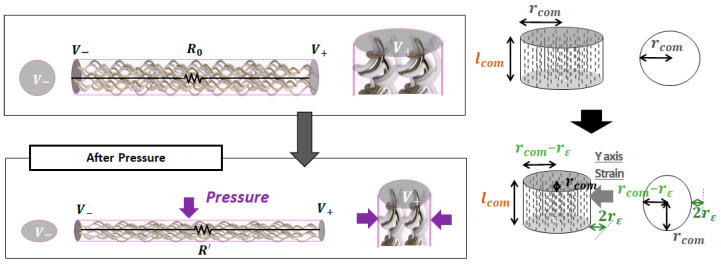
Resistance change (under pressure) when considering the characteristics of the *Fe-sp* yarns as a cylindrical composite material.

The resistance changes in the second half of the deformation due to the dispersion of Fe and Ni of the *Fe-sp* yarns (Figure 3). Considering these yarns as a cylindrical composite material, the yarn’s volume ratio (Q) decreases, and the distance (d) between these fibers reduces. The number and weight of conductive fibers are constant. In this stage, the resistance change of *Fe-sp* and *Wt-Cfa* can be expressed by Equations (10) and (11).

(10) $$\begin{array}{l} R\left(\epsilon \right)=R_{\left(Fe-sp\right)}^{\prime }=\left(\frac{l_{thrd}}{{\left(d_{thrd}\right)}^{2}\pi }\sqrt[{3}]{\frac{V_{fib}\left(1-\frac{\sigma }{E_{pll}}\right)}{Q_{0}}}\right)[\left\{\rho_{fib}\frac{l_{fib}}{{\left(\frac{d_{fib}}{2}\right)}^{2}\pi }\right\}\\ \;\;\;\;\;\;\;+\left\{\frac{4.94\left\{10^{23}e^{\left(4.1\lambda \sqrt{\lambda }\right)}\right\}}{\left(\frac{d_{fib}}{2}\right)\left(\frac{l_{fib}}{2}\right)\sqrt{\lambda }}\right\}\left(\frac{\sqrt{2}}{2}\left(\sqrt[{3}]{\frac{V_{fib}}{Q_{0}}}-2r_{fib}\right)+\frac{\sqrt{2}}{2}\left(\sqrt[{3}]{\frac{V_{fib}\left(1-\frac{\sigma }{E_{pll}}\right)}{Q_{0}}}-2r_{fib}\right)\right)] \end{array}$$

(11) $$\begin{array}{l} R\left(\sigma \right)=[\frac{\sin\left\{\tan^{-1}\left(DC_{hfthrd}T\omega_{thrd}\right)\right\}}{\cos\left[\sin^{-1}\left\{\left(1-\left(\frac{\sigma \tan\left\{\tan^{-1}\left(DC_{hfthrd}T\omega_{thrd}\right)\right\}}{n_{hfthrd}E_{Ag-tw}\pi DC_{hfthrd}^{2}(cos^{2}\left(\tan^{-1}DC_{hfthrd}T\omega_{thrd}\right))}\right)\right)\sin\left\{\tan^{-1}DC_{hfthrd}T\omega_{thrd}\right\}\right\}\right]}\\ \;\;\;\;\;\;\;*D\frac{C_{hfthrd}}{2}T\omega_{thrd}]R_{0\left(Wt-Cfa\right)}\\ \;\;\;\;\;\;\;\;\;\;\;\;\;\;\mathrm{but},\;\epsilon <\frac{DC_{hfthrd}-d_{hfthrd}}{d_{hfthrd}} \end{array}$$

**Figure 4:**
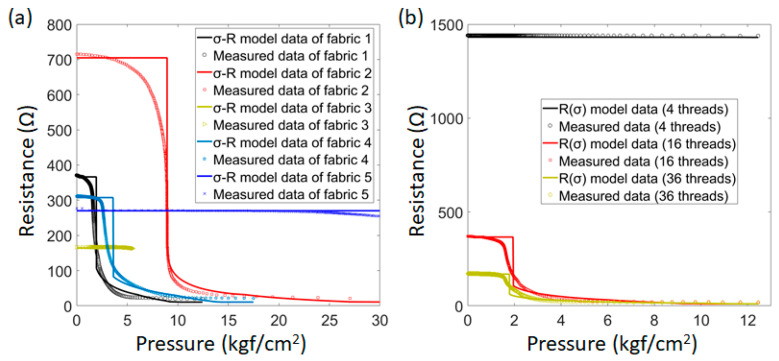
(**a**) The measured and calculated values (σ-R model) of the different fabrics, and (**b**) the measured and calculated values (R(σ) model) of the different sensing yarns (*Fe-sp*).

The potential of the developed model is demonstrated in Figure 4. There are relationships between the resistance change and the compression, consisting of Ag-coated twisted yarns, Fe staple-fiber spun yarns, and the structure of the spacer fabric. We obtained that the measured values and the calculated values from the model are consistent at the high-pressure stage.

There are some differences between the model and the measured result in the initial low-pressure stage. It is impossible to predict the pressure range before the 2nd contact accurately. The reason may be the interaction between the conductive materials (Ag and Fe). We suggest one solution that uses only Ag-coated for both the twisted yarns and the spun yarns. However, this requires further research. This paper is part of a wider study of the effects of textile and structural elements on wearable sensors that were overlooked in other studies. Building on the authors’ previous publication, a more complete understanding of the factors that affect textile pressure sensors has been obtained.

### 3.4. Resonant Frequencies and Matrix Touch Sensor

**Figure 5:**
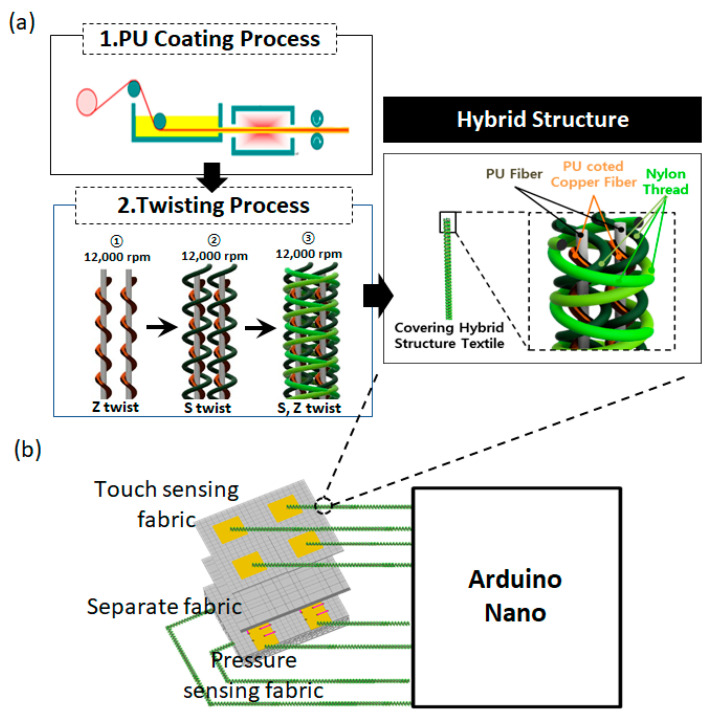
(**a**) Hybrid twisted Cu fibers (TCFs) and (**b**) structure of the matrix touch sensor.

Twisted Cu fibers are manufactured from a plating process and are used to transmit signals from sensors to an integration device. As described in Figure 5a, metal yarns are coated by PU at the speed of 0.54 cm/s, and a curing temperature of 120 °C. A twisted Cu fiber (TCF) is Z-twisted with 560D PU filament. Multi-twisted Cu fibers (TCFs) are S-twisted with 140D nylon filament. The final yarn is a composite seal structure featuring both Z-twist and S-twist.

**Figure 6:**
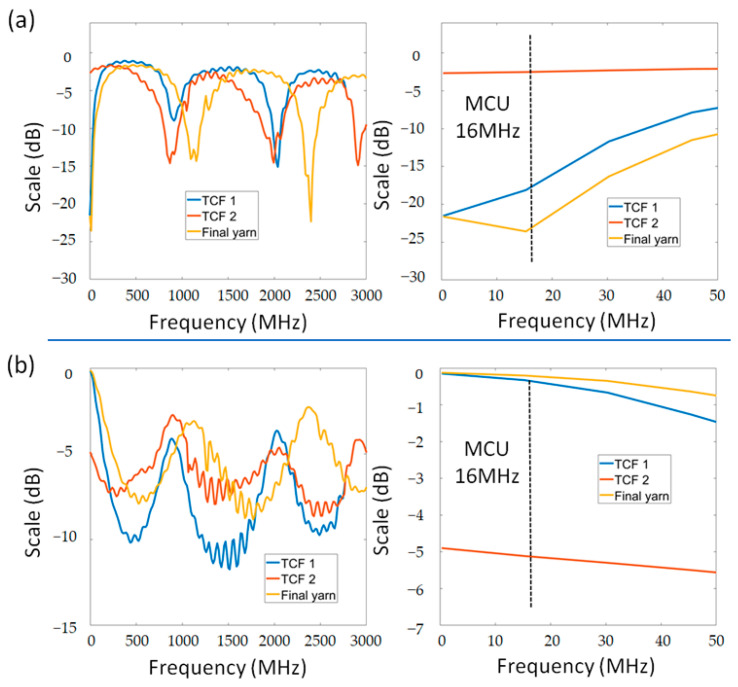
(**a**) Transmission and (**b**) reflection characteristics of the different structure strands (TCF 1, TCF 2, and final strand).

To demonstrate the ability of the sensing structure, we deploy a simple pressure matrix touch sensor (2 × 2) in Figure 5b, including two *Wt-Cfa* layers at the top and bottom of Wp-Cfa. There are 16 sensing lines (*Fe-sp*) with an inclination of 5.2° on the x/y axis. The touching signal is transmitted from the sensors to an integrated circuit device (Arduino Nano) by the TCFs. Through a network analyzer (E5061B Agilent), we obtained the resonant frequencies of the TCFs from 300 Mhz to 500 Mhz. The total change of the reflection characteristics is about 24 dB at 16 Mhz (Figure 6). These values are suitable for the Arduino Nano board.

**Figure 7:**
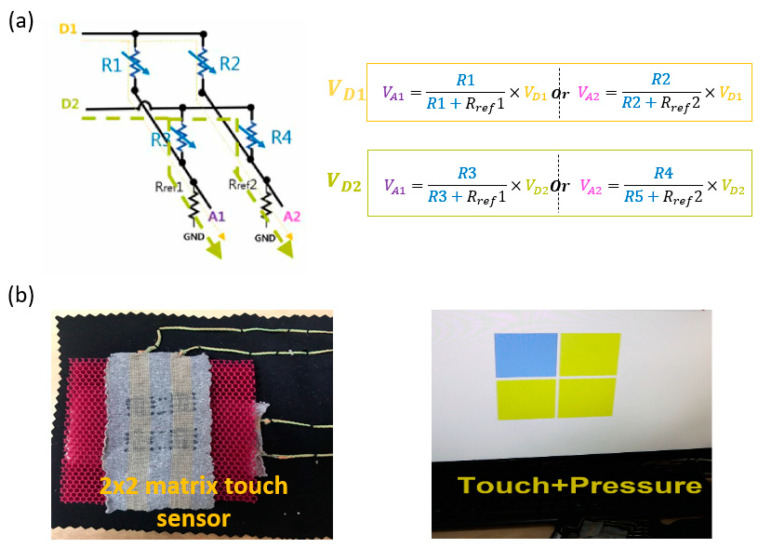
(**a**) Cross-power measurement method and (**b**) matrix touch sensor (2 × 2).

As shown in Figure 7, matrix resistors can measure a force by the voltage change (cross-power method) at two digital pins (D1, D2). Analog values (A1, A2) are calculated from the distributed voltages. As expected, the system can detect a touch/pressure position and show it on a monitor.

## 4. Conclusions

This paper developed a pressure textile sensor from the knitting process and the textile elements, including warp-knitted spacer fabrics, Ag-coated twisted yarns, and Fe staple-fiber spun yarns. The resistance change is due to the compression characteristics of the textile structure, the buckling, and the contacts. Herein, the effects of Fe staple-fiber spun yarns are analyzed in detail. In addition, the textile electrical properties could be predicted and evaluated by an R-ε correlation model. This is a potential step in the development of wearable devices that need high precision. We also deployed a matrix touch sensor as a demonstration in an actual application.

The sensor is expected to apply to human–machine interfaces, robots, or home e-textiles. In addition, some touch/pressure integrated samples have been designed for interior automobile systems that can control music mode, brightness mode, and even move the car seat position.

## Figures and Tables

**Table 1:** Characteristics of the Wp-Sfa fabrics from optical microscope image analysis.

		The 1st Fabric	The 2nd Fabric	The 3rd Fabric	The 4th Fabric	The 5th Fabric
***h***		5502.4 μm	10,743.5 μm	2371.8 μm	4587.3 μm	4014.9 μm
$d_{pll}$		63.7 μm	164.5 μm	46.2 μm	57.3 μm	253.4 μm
$l_{pll}$		6625.7 μm	14,086.5 μm	3538.7 μm	5523.1 μm	4606.9 μm
$n_{pll}$		917 ea/cm^2^	86 ea/cm^2^	1795 ea/cm^2^	1417 ea/cm^2^	8.83 ea/cm^2^
$E_{pll\left(ex\right)}$		148.7 kgf/cm^2^	138 kgf/cm^2^	175.1 kgf/cm^2^	149.5 kgf/cm^2^	143 kgf/cm^2^
**Pylon geometry rescue**	**R**	3295.5 μm	6012.5 μm	1202 μm	3321.5 μm	3229.1 μm
	**|AB**	5581.7 μm	11,027.7 μm	2368.2 μm	4820.4 μm	4148.6 μm
	**∠α**	9.6°	10.2°	6.5°	22.2°	9.9°
	**∠β**	115.3°	134.3°	168.6°	95.1°	81.8°
	**|DB**	900.7 μm	1991 μm	262.3 μm	1795 μm	722.5 μm

## Data Availability

Data is contained within the article or Supplementary Material.

## Supplementary Materials

The following supporting information can be downloaded at: https://www.mdpi.com/article/10.3390/s22093152/s1. Figure S1. SEM and XRF pictures of (a) Ag yarns and (b) Fe yarns. Figure S2. (a) Geometric structure and (b) the side view of the spacer fabrics 1–5. Figure S3. (a) Universal testing machine (UTM), (b) the resistance change with no-slope at the sensing yarns, (c) the resistance change with x-slope at the sensing yarns, and (d) the resistance change with x/y-slope at the sensing yarns.

## Abbreviations

h	Thickness of fabric
$n_{thrd}$	Number of the stitched *Fe-sp*
$R_{0}$	Initial resistance of the *Fe-sp*
$d_{pll}$	Diameter of the pile yarns
R	Curvature radius of the pile yarns
$l_{pll}$	Length of the pile yarn
$n_{pll}$	Number of the pile yarn
∠α	Slope angle of the pile yarn curve
∠β	Central angle of the pile yarn curve
|AB	Length of the AB line
|DB	Length of the DB
$E_{pll\left(ex\right)}$	Tension modulus
$d_{thrd}$	Diameter of the *Fe-sp*
$l_{thrd}$	Length of the *Fe-sp*
$Q_{0}$	Volume fraction of conductive staple fiber
$V_{fib}$	Volume of the conductive staple fiber
$d_{fib}$	Diameter of the conductive staple fiber
$l_{fib}$	Length of the conductive staple fiber
λ	Energy barrier
