# Supplementary material for: Effects of Fe Staple-Fiber Spun-Yarns and Correlation Models on Textile Pressure Sensors
Source: Sensors (Basel). 2022 Apr 20;22(9):3152. doi: 10.3390/s22093152 (PMC9100205; doi:10.3390/s22093152)
Supplement: Supplementary file 1 [file sensors-22-03152-s001.zip › sensors-1606826-supplementary.pdf]

Supplementary Materials

Effects of Fe Staple-Fiber Spun-Yarns and Correlation Models on Textile Pressure Sensors

Minki Choi, Chi Cuong Vu and Jooyong Kim

The Ag-tw has two bundles of twisted yarns, and each bundle has multi-microfilaments. Using X-ray method (XRF), we obtained that the percentage weight of Ag is about 5.88% on the entire yarn (Figure S1a).

$$Ag: Nylon = 5.88: 94.12\ (wt\%) \rightarrow 0.64: 99.36\ (V\%)$$

$$Ag_{Density} = 10.5\ g/cm^3\ ,\ Nylon_{Density} = 1.05\ \ g/cm^3$$

$$(Nylon + Ag): (Nylon) = 100: 99.36\ (V\%) = (d_{fib})^2: (d_{fib} - t_{Ag})^2$$

$$\text{So, } t_{Ag} \approx 22\ nm \text{ (the coating thickness of Ag)}$$

In addition, the Fe yarn is composed of Fe, Ni, and PET with percentage weights of 16.57%, 3.27%, 80.16%, respectively (Figure S1b).

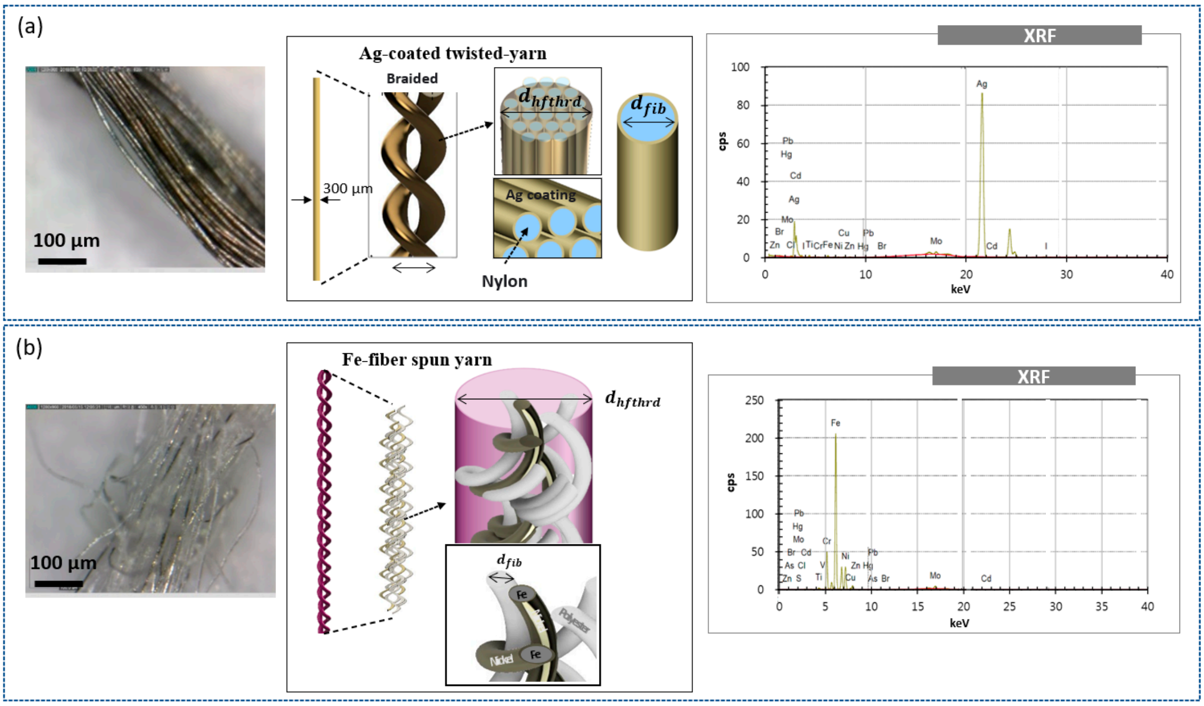

Figure S1. SEM and XRF pictures of (a) Ag yarns and (b) Fe yarns.

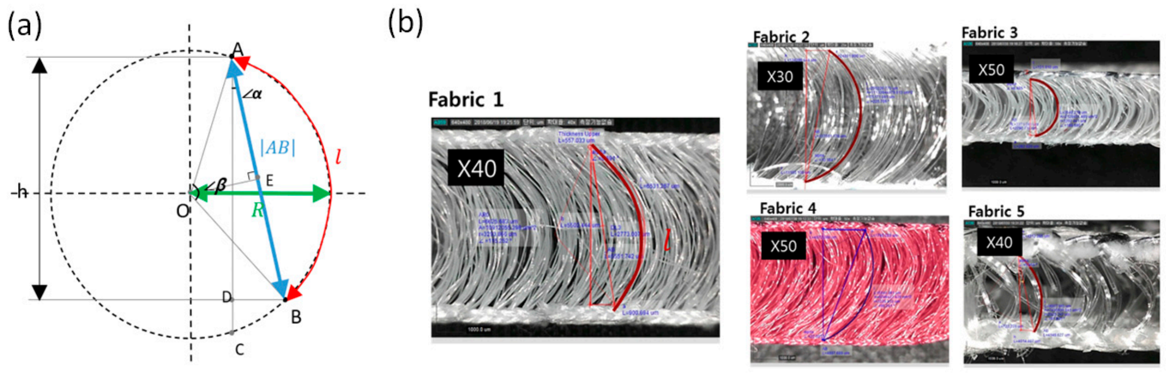

Figure S2. (a) Geometric structure and (b) the side view of the spacer fabrics 1–5.

As shown in Figure S2, the spacer fabrics are composed of multi polyester monofilaments and knitted together. On the diagram, h is the thickness of the fabric, R is the curvature radius of the pile yarns, $\angle\alpha$ is the slope angle of the pile yarn curve, $\angle\beta$ is the central angle of the pile yarn curve, and l is the length of the pile yarn.

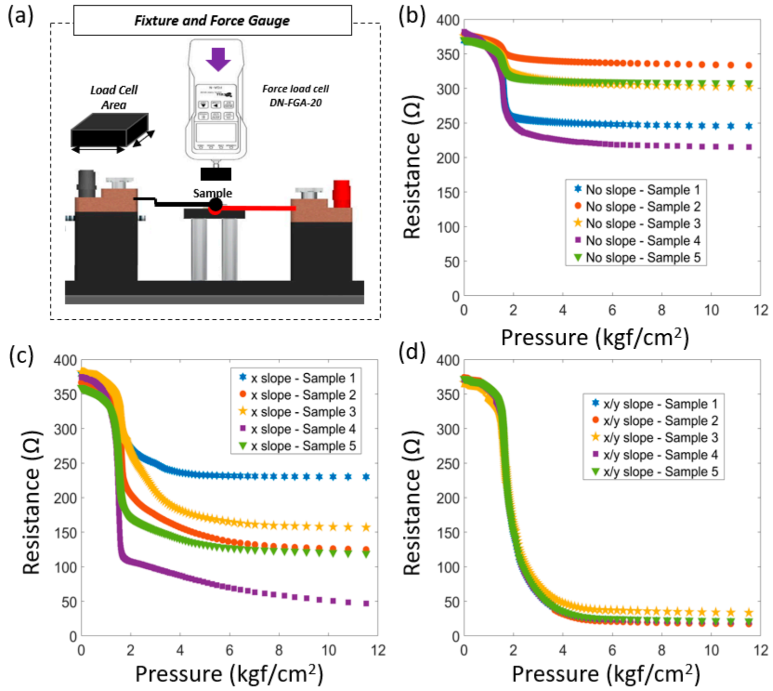

Figure S3. (a) Universal testing machine (UTM), (b) the resistance change with no-slope at the sensing yarns, (c) the resistance change with x-slope at the sensing yarns, and (d) the resistance change with x/y-slope at the sensing yarns.
